# Comparative mitochondrial genome analysis of the Mongolian redfin, *Chanodichthys mongolicus* (Xenocyprididae) from China reveals heteroplasmy

**DOI:** 10.1080/23802359.2021.1961627

**Published:** 2021-08-10

**Authors:** Kai Liu, Xiao-yu Feng, Heng-Jia Ma, Nan Xie

**Affiliations:** Institute of Fishery Science, Hangzhou Academy of Agricultural Sciences, Hangzhou, China

**Keywords:** *Chanodichthys mongolicus*, comparative mitochondrial genomes, heteroplasmy

## Abstract

This study determined the mitochondrial genome (mitogenome) of *Chanodichthys mongolicus* from China's Qiantang River and analyzed its phylogenetic history in the Subfamily Cultrinae. Next-generation sequencing was used to obtain the mitogenome of *C. mongolicus*, GenBank Accession Number MZ032228. The mitochondrial genome length of *C. mongolicus* from China's Qiantang River is 16,622 bp. The genome contains 13 protein-coding genes, 22 transfer RNAs, two ribosomal RNAs, and two central noncoding regions (the control region and the origin of light strand replication). Based on BLAST comparisons, the sequence identity of *C. mongolicus* MZ032228 from China's Qiantang River was 99.84% to that of *Ancherythroculter wangi* MG783573 from China's Nei River, 99.75% to *C. mongolicus* AP009060 from Russia's Black River. The phylogenetic analysis is consistent with BLAST comparisons in confirming that *A. wangi* MG783573 and *C. mongolicus* MZ032228 show a high genetic similarity. This study also confirms mitochondrial DNA heteroplasmy in *C. mongolicus* for the first time and documents 35 heterogeneous loci that were detected.

## Introduction

The Mongolian redfin *Chanodichthys mongolicus* (Basilewsky 1855) is an economically important species of ray-finned fish classified in the genus *Chanodichthys*. The species is widely distributed in many reservoirs and lakes of China (Chen 1998). The Mongolian redfin was once recognized as the main aquaculture species in China (Xie et al. 2019). However, due to overfishing, habitat destruction, and water pollution, *C. mongolicus* stocks in natural waters have seriously declined. In recent years, the artificial propagation and release of the Mongolian redfin have been carried out in some regions of China (Xie et al. 2019). At present, genetic studies on *C. mongolicus* are relatively few. Although some researchers have reported the mitochondrial genome (mitogenome) of *C. mongolicus* (Saitoh et al. 2006; Tong et al. 2014; Wei et al. 2016), comparative mitogenomic analyses of *C. mongolicus* from the different geographical populations have not been reported. In this study, the mitogenome of *C. mongolicus* from China's Qiantang River was determined for the first time. A comparative analysis of the mitogenomes among *C. mongolicus* was undertaken. Other culter fishes are also included in the analyses to confirm the phylogenetic position of *C. mongolicus* in the Cultrinae. In addition, mitochondrial DNA (mtDNA) heteroplasmy in *C. mongolicus* is investigated and documented for the first time.

## Materials and methods

The Mongolian redfin was harvested from Qiantang River (120°10′13.15″E, 30°07′11.22″N) and deposited at the National Original Breeding Farm of black Amur bream from Qiantang River (120°07′21.99″E, 30°08′35.53″N, www.hznky.com, Kai Liu, liukai0106@email.cn). The total genomic DNA from the fin tissue (assigned as MGB201906) was extracted by the phenol-chloroform extraction method (Green and Sambrook 2012). After the genomic DNA was quantified, the DNA was sonicated using a Covaris M220. The sheared DNA fragments were purified and used to construct a sequencing library with the following steps: end-polished, A-tailed, and ligated with the full-length adaptor for Illumina sequencing with further PCR amplification (Quail et al. 2008). Finally, the purified DNA fragments were subjected to next-generation sequencing (NGS). The NGS was performed by Personal Gene Technology CO., Ltd (Shanghai, China). The mitochondrial genome of the Mongolian redfin was obtained by sequence assembly on the NGS data using GetOrganelle ver. 1.7.3.5 (Jin et al. 2020) with average base coverage of 151.8. The accession was registered GenBank under accession number MZ032228. The annotation process was completed using the MITOFISH prediction server (Iwasaki et al. 2013). Mitogenome sequence consistency was compared by CGView Comparison Tool ver. 1.0 (Grant et al. 2012). Detection of mitochondrial heteroplasmy in *C. mongolicus* was performed by NOVOPlasty ver. 4.3.1 (Dierckxsens et al. 2020) and visualized by Circos ver. 0.69.8 (Krzywinski et al. 2009). The phylogenetic analysis was performed by IQ-TREE ver. 2.1.3 with the complete mitogenomes, under the TPM2 + F + I + G4 substitution model (Minh et al. 2020). Mitogenome sequences were firstly aligned using the MAFFT component of UGENE ver. 38.1 (Okonechnikov et al. 2012) and then trimmed using trimAl ver. 1.2rev57 (Capella-Gutierrez et al. 2009). SH-aLRT test values and Ultrafast Bootstrap values were given in percentages (Hoang et al. 2018).

## Results and discussion

**Figure 1.**
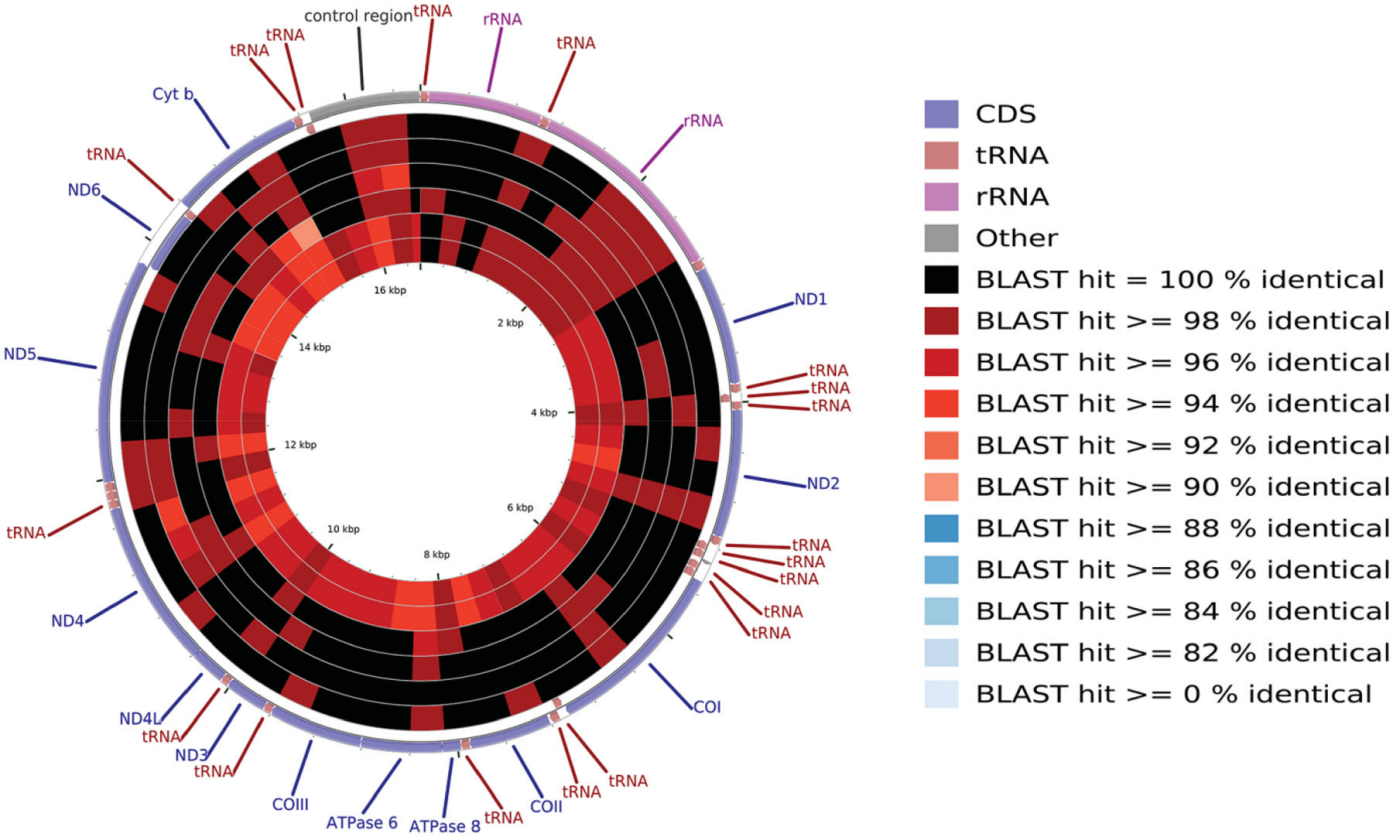
A BLAST-based pie chart of the sequence identity of the mitochondrial genomes of *Chanodichthys mongolicus*, *Ancherythroculter wangi,* and *Ancherythroculter kurematsui* from the different geographical populations. The circle from outside to inside represents *Chanodichthys mongolicus* MZ032228 from China's Qiantang River, *Ancherythroculter wangi* MG783573 from China's Nei River, *Chanodichthys mongolicus* AP009060 from Russia's Black River, *Chanodichthys mongolicus* KF826087, *Chanodichthys mongolicus* KC701385 from China's Heilong River, *Ancherythroculter kurematsui* KU234534 from China's Jialing River, and *Ancherythroculter wangi* MG575902 from China's Qi River

The mitogenome of *C. mongolicus* from China's Qiantang River contains 22 tRNAs, two ribosomal RNAs, 13 protein-coding genes (PCGs), and two central non-coding regions. Most genes of *C. mongolicus* are encoded on the heavy strand (H-strand) except for *ND*6 and eight tRNA, which are encoded on the light strand (L-strand). Within the genome, all 13 PCGs include the standard start codon ATG except for *CO*1, which is initiated with GTG. However, the stop codons of the 13 PCGs differ, these terminating with TAG, TAA, TA– or T––. The origin of light strand replication (OL), which extends up to 31 nucleotides, is identified in the WANCY region. The second non-coding region, the control region (D-loop), is 938 bp in length and located between tRNA-Pro and tRNA-Phe. The complete mitogenome of *C. mongolicus* from China's Qiantang River is 16,622 bp. The length is the same as two other *C. mongolicus* mitogenomes (Saitoh et al. 2006; Wei et al. 2016), and one bp longer than the mitogenome of *C. mongolicus* from China's Heilong River (Tong et al. 2014). Based on the comparison of BLAST results (Figure 1), the sequence identity of *C. mongolicus* MZ032228 from China's Qiantang River is 99.84% to that of *Ancherythroculter wangi* MG783573 from China's Nei River (Zou et al. 2018), 99.75% to *C. mongolicus* AP009060 from Russia's Black River, 99.70% to *C. mongolicus* KF826087, 99.49% to *C. mongolicus* KC701385 from China's Heilong River, 97.18% to *Ancherythroculter kurematsui* KU234534 from China's Jialing River (Wang et al., 2016), and 97.17% to *A. wangi* MG575902 from China's Qi River (Yan et al. 2017).

**Figure 2.**
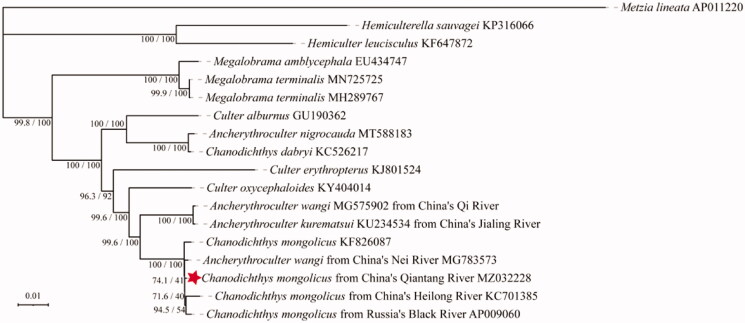
Phylogenetic tree of *Chanodichthys mongolicus* inferred using the maximum likelihood method based on the mitochondrial genome. Values are shown at each node of the tree, correspond to the SH-aLRT test values and Ultrafast Bootstrap value given in percentages.

The phylogenetic analysis fully resolved *C. mongolicus* in a clade with four other published mitogenomes (Figure 2). These included the three sequences of *C. mongolicus* cited above as well as the mitogenome of *A. wangi* MG783573. Within the clade, the SH-aLRT and Ultrafast Bootstrap values on some of the nodes were low. The *A. wangi* MG783573 sequence did not cluster with the other *Ancherythroculter* sequences, including another *A. wangi* MG575902 (Yan et al. 2017). This finding appears to point to an error or misidentification of that MG783573 specimen.

**Figure 3.**
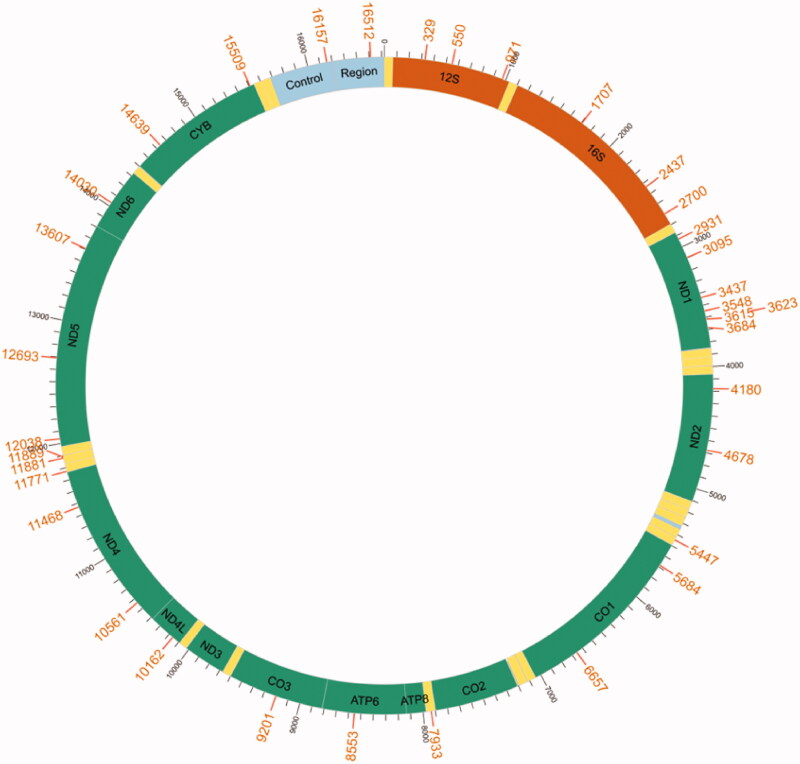
The detected heterogeneous loci are indicated by their position on the mitochondrial genome of *Chanodichthys mongolicus.*

mtDNA heteroplasmy in humans is common (Stewart and Chinnery 2015) and has been observed and studied in other vertebrates (Gajic et al. 2016; Gorkhali et al. 2016; Huang et al. 2019; Sriboonlert and Wonnapinij 2019). In *C. mongolicus*, 35 heterogeneous loci were detected with an average value of allele frequency and raw depth of 0.02 and 176.2, respectively (Figure 3). The heterogeneous loci are mainly located in PCGs and are also present in the D-loop, some tRNAs, and rRNAs. Polymorphic sites in the PCGs resulted in 18 nonsynonymous and three synonymous substitutions. The *ND*1 gene showed the most heterogeneous loci, with seven loci. Based on the work by Wetjen et al. (2017), the D-loop region was the primary region of mtDNA heterogeneity in fish detected by PCR methods. mtDNA heteroplasmy has also been identified in many gene locations in Loricarioidei using NGS data (Moreira et al. 2017). Heteroplasmic positions were found in almost all Loricarioidei species, except one. Mutations were scattered across the mitogenomes of Loricarioidei. The result of mtDNA heteroplasmy detected in Loricarioidei is similar to this study.

## Data Availability

The genome sequence data that support the findings of this study are openly available in GenBank of NCBI at [https://www.ncbi.nlm.nih.gov] (https://www.ncbi.nlm.nih.gov/) under the accession no. MZ032228. The associated BioProject, SRA, and BioSample numbers are PRJNA732476, SRX10977011, and SAMN19321491, respectively.

## References

[CIT0001] Capella-Gutierrez S, Silla-Martinez JM, Gabaldon T. 2009. trimAl: a tool for automated alignment trimming in large-scale phylogenetic analyses. Bioinformatics. 25(15):1972–1973. 19505945 10.1093/bioinformatics/btp348 PMC2712344

[CIT0002] Chen YY. 1998. Fauna Sinica: Osteichthyes Cypriniformes II. Beijing: Science Press.

[CIT0003] Dierckxsens N, Mardulyn P, Smits G. 2020. Unraveling heteroplasmy patterns with NOVOPlasty. NAR Genom Bioinform. 2(1):lqz011. 33575563 10.1093/nargab/lqz011 PMC7671380

[CIT0004] Gajic B, Stevanovic J, Radulovic Z, Kulisic Z, Vejnovic B, Glavinic U, Stanimirovic Z. 2016. Haplotype identification and detection of mitochondrial DNA heteroplasmy in Varroa destructor mites using ARMS and PCR-RFLP methods. Exp Appl Acarol. 70:287–297. 27631761 10.1007/s10493-016-0086-6

[CIT0005] Gorkhali NA, Jiang L, Shrestha BS, He XH, Junzhao Q, Han JL, Ma YH. 2016. High occurrence of mitochondrial heteroplasmy in Nepalese indigenous sheep (*Ovis aries*) compared to Chinese sheep. Mitochondrial DNA Part A. 27(4):2645–2647. 10.3109/19401736.2015.1041134 26084311

[CIT0006] Grant JR, Arantes AS, Stothard P. 2012. Comparing thousands of circular genomes using the CGView Comparison Tool. BMC Genomics. 13:202. 22621371 10.1186/1471-2164-13-202 PMC3469350

[CIT0007] Green MR, Sambrook J. 2012. Molecular cloning: a laboratory manual, 4th ed. New York: Cold Spring Harbor Laboratory Press.

[CIT0008] Hoang DT, Chernomor O, von Haeseler A, Minh BQ, Vinh LS. 2018. UFBoot2: improving the ultrafast bootstrap approximation. Mol Biol Evol. 35(2):518–522. 29077904 10.1093/molbev/msx281 PMC5850222

[CIT0009] Huang Y, Lu W, Ji J, Zhang X, Zhang P, Chen W. 2019. Heteroplasmy in the complete chicken mitochondrial genome. PLOS One. 14(11):e0224677. 31703075 10.1371/journal.pone.0224677 PMC6839896

[CIT0010] Iwasaki W, Fukunaga T, Isagozawa R, Yamada K, Maeda Y, Satoh TP, Sado T, Mabuchi K, Takeshima H, Miya M, et al. 2013. MitoFish and MitoAnnotator: a mitochondrial genome database of fish with an accurate and automatic annotation pipeline. Mol Biol Evol. 30(11):2531–2540. 23955518 10.1093/molbev/mst141 PMC3808866

[CIT0011] Jin JJ, Yu WB, Yang JB, Song Y, dePamphilis CW, Yi TS, Li DZ. 2020. GetOrganelle: a fast and versatile toolkit for accurate de novo assembly of organelle genomes. Genome Biol. 21(1):241. 32912315 10.1186/s13059-020-02154-5 PMC7488116

[CIT0012] Krzywinski M, Schein J, Birol I, Connors J, Gascoyne R, Horsman D, Jones SJ, Marra MA. 2009. Circos: an information aesthetic for comparative genomics. Genome Res. 19(9):1639–1645. 19541911 10.1101/gr.092759.109 PMC2752132

[CIT0013] Minh BQ, Schmidt HA, Chernomor O, Schrempf D, Woodhams MD, von Haeseler A, Lanfear R. 2020. IQ-TREE 2: new models and efficient methods for phylogenetic inference in the genomic era. Mol Biol Evol. 37(5):1530–1534. 32011700 10.1093/molbev/msaa015 PMC7182206

[CIT0014] Moreira DA, Buckup PA, Furtado C, Val AL, Schama R, Parente TE. 2017. Reducing the information gap on Loricarioidei (Siluriformes) mitochondrial genomics. BMC Genomics. 18(1):345. 28472937 10.1186/s12864-017-3709-3 PMC5418769

[CIT0015] Okonechnikov K, Golosova O, Fursov M, UGENE team 2012. Unipro UGENE: a unified bioinformatics toolkit. Bioinformatics. 28(8):1166–1167. 22368248 10.1093/bioinformatics/bts091

[CIT0016] Quail MA, Kozarewa I, Smith F, Scally A, Stephens PJ, Durbin R, Swerdlow H, Turner DJ. 2008. A large genome center's improvements to the Illumina sequencing system. Nat Methods. 5(12):1005–1010. 19034268 10.1038/nmeth.1270 PMC2610436

[CIT0017] Saitoh K, Sado T, Mayden RL, Hanzawa N, Nakamura K, Nishida M, Miya M. 2006. Mitogenomic evolution and interrelationships of the Cypriniformes (Actinopterygii: Ostariophysi): the first evidence toward resolution of higher-level relationships of the world's largest freshwater fish clade based on 59 whole mitogenome sequences. J Mol Evol. 63(6):826–841. 17086453 10.1007/s00239-005-0293-y

[CIT0018] Sriboonlert A, Wonnapinij P. 2019. Comparative mitochondrial genome analysis of the firefly, *Inflata indica* (Coleoptera: Lampyridae) and the first evidence of heteroplasmy in fireflies. Int J Biol Macromol. 121:671–676. 30339996 10.1016/j.ijbiomac.2018.10.124

[CIT0019] Stewart JB, Chinnery PF. 2015. The dynamics of mitochondrial DNA heteroplasmy: implications for human health and disease. Nat Rev Genet. 16(9):530–542. 26281784 10.1038/nrg3966

[CIT0020] Tong GX, Kuang YY, Geng LW, Xu W, Yin JS. 2014. Mitochondrial DNA sequence of Mongolian redfin (*Chanodichthys mongolicus*). Mitochondrial DNA. 25(5):407–409. 23795832 10.3109/19401736.2013.803539

[CIT0021] Wang GJ, Zheng ZL, Yu EM, Xie J, Wei N, Wu JR, Li JS. 2016. The complete mitochondrial genome of *Ancherythroculter kurematsui* (Cypriniformes: Cyprinidae). Mitochondrial DNA Part B. 1(1):630–631. 33473577 10.1080/23802359.2016.1214547 PMC7800160

[CIT0022] Wei M, Yang Y, Yu P, Pan D, Wan Q. 2016. The complete mitochondrial genome of Mongolian redfin, *Chanodichthys mongolicus* (Cypriniformes: Cyprinidae): genome description and related phylogenetic analyses. Mitochondrial DNA Part A. 27(1):20–21. 10.3109/19401736.2013.867440 24438306

[CIT0023] Wetjen M, Cortey M, Vera M, Schmidt T, Schulz R, García-Marín J-L. 2017. Occurrence of length polymorphism and heteroplasmy in brown trout. Gene Rep. 6:1–7.

[CIT0024] Xie JY, Yan Y, Yang YH, Lin SQ. 2019. Analysis on genetic structure of *Chanodichthys mongolicus* populations by mitochondrial COI gene sequences. Freshw Fish. 49(03):3–7.

[CIT0025] Yan T, Wang X, Zhang S, He L, He Z. 2017. The complete mitochondrial DNA of *Ancherythroculter wangi*. Mitochondrial DNA Part B. 3(1):19–20. 33490483 10.1080/23802359.2017.1413320 PMC7800375

[CIT0026] Zou Y, Zhang J, Xie M, Zhang T, Deng Q, Wen Z. 2018. The complete mitochondrial genome of *Ancherythroculter wangi* and its phylogeny. Mitochondrial DNA Part B. 3(1):276–277. 33474140 10.1080/23802359.2018.1443035 PMC7799608

